# Designing a Clinical Trial Protocol about the Impact of Family-Based Multimedia Education Based on Telephone Tracking (Tele Nursing) to Improve the Quality of Life and Self-Efficacy in Patients with Myocardial Infarction

**DOI:** 10.29337/ijsp.146

**Published:** 2021-05-28

**Authors:** Shirin Madadkar Dehkordi, Forogh Okhovat, Zohreh Karimiankakolaki

**Affiliations:** 1 Department of Nursing, Shahrekord Branch, Islamic Azad University, Shahrekord, Iran; 2 Ph.D Health Education and Promotion, Department of Health, Shahrekord Branch, Islamic Azad University, Shahrekord, Iran

**Keywords:** Education, Family- Centered, Tele Nursing, Quality Of Life, Self-Efficacy, Myocardial Infarction

## Abstract

**Background:**

Provision of education to a person with myocardial infarction and an active family member, who takes care of the patient can prevent or delay the onset of the disease. Telephone tracking is a very useful and inexpensive way to assess the patients’ needs and help them with their care problems. This clinical trial (interventional) protocol was conducted over the impact of family-based multimedia education based on the telephone tracking (tele-nursing) to improve the quality of life and self-efficacy in patients with myocardial infarction.

**Methods:**

Two phases are identified to design this study; the first phase includes designing a curriculum by investigating various studies and the panel of experts’ opinions. This phase will be conducted in the form of multimedia training and telephone contact. Multimedia training (including audio, video, image, and animation) over the patients’ lifestyle, nutrition, and care will be conducted through a one-day workshop in 2-3 hours for patients and one of their active family members. The active family member is defined as the primary caregiver, who spends more time with the patient. The educational course will be conducted at a coordinated date and time in the ward where the patients are hospitalized. The researcher will make telephone calls as the educational intervention and continue the follow-ups once a week for one month. The second phase of the intervention will contain a pre/post-test design along with application of Minnesota quality of life and Scherer general self-efficacy standard questionnaires in the intervention (with training) and control (without training) groups. The target participants will include all patients (and their active family members) admitted to CCUs of hospitals affiliated to Shahrekord University of Medical Sciences.

**Discussion:**

The present study provides useful data for designing a family-based multimedia educational intervention using the telephone-tracking method (tele-nursing) to improve the quality of life and self-efficacy in patients with myocardial infarction. It can also reduce their medical and treatment costs. The strategies of this program could be important and cost effective, and therefore we hope that the success of such a program is a step forward in improving cardiovascular patient’s health status.

**Highlights:**

- Family-based multimedia educational (tele-nursing) to improve the quality of life and self-efficacy in patients with myocardial infarction.
- Family-based multimedia educational (tele-nursing) can also reduce their medical and treatment costs.
- The program is a step forward in improving cardiovascular patient’s health status.

## Background

Cardiovascular diseases are among the chronic illnesses and leading causes of death in the world. By 2020, cardiovascular diseases are estimated to be the first cause of disability on the world’s list of debilitating diseases [7]. Among the ischemic heart diseases, myocardial infarction is one of the most common and dangerous diseases in industrialized countries [1]. Myocardial infarction is ischemic necrosis of the cardiac myocytes that occurs due to a lack or loss of blood flow [6]. In the United States, about 5 million people develop cardiovascular diseases each year, which kills 285,000 people annually [13]. In Iran, cardiovascular diseases account for more than 46% of the total mortality rate, half of which is due to myocardial infarction [11]. Widespread expansion of the myocardial infarction causes complications such as congestive heart failure, acute pulmonary edema, cardiogenic shock, and mortality. It also imposes high care and treatment costs on patients [12]. Myocardial infarction is a non-infectious disorder that affects many aspects of life in patients and their family members, including their quality of life and self-efficacy [13]. In other words, chronic nature of the disease exacerbates the patients’ inability to meet their own needs. Failure to follow the health care diets is an important factor in increasing the odds of complications, mortality rates, and health care costs, while decreasing the quality of life and self-efficacy. Quality of life includes physical, psychological, and social dimensions as well as the patient’s personal feeling of health [3,10]. Provision of education to a person with myocardial infarction and an active family member, who takes care of the patient can prevent or delay the onset of the disease [2]. Family-based education involves active presence of a family member in identifying the patients’ needs and training them, since this type of education is based on the fact that occurrence of a disease in an individual enters all the family members into the disease cycle [14]. Multimedia software education, as one of the new educational methods, has affected all aspects of human life with the increasing advent of computers, information, and communication [5]. Telephone tracking is a very useful and inexpensive way to assess the patients’ needs and help them with their care problems [4]. Post-discharge telephone contact is helpful in identifying and modifying the care gaps that may occur after discharge [8]. This clinical trial (interventional) protocol was conducted over the impact of family-based multimedia education based on the telephone tracking (tele-nursing) to improve the quality of life and self-efficacy in patients with myocardial infarction.

## Methods/design

Two phases are identified to design this study; the first phase includes designing a curriculum by investigating various studies and the panel of experts’ opinions. This phase will be conducted in the form of multimedia training and telephone contact. Multimedia training (including audio, video, image, and animation) over the patients’ lifestyle, nutrition, and care will be conducted through a one-day workshop in 2–3 hours for patients and one of their active family members. The active family member is defined as the primary caregiver, who spends more time with the patient. The educational course will be conducted at a coordinated date and time in the ward where the patients are hospitalized. The researcher will make telephone calls as the educational intervention and continue the follow-ups once a week for one month. The time of telephone calls will be coordinated with the patients and their companions. The tele-communication contents will include verbal encouragement about health behaviors related to the educational and workshop contents. The research team will finalize the contents based on their priorities. The second phase of the intervention will contain a pre/post-test design along with application of Minnesota quality of life and Scherer general self-efficacy standard questionnaires in the intervention (with training) and control (without training) groups.

## Aim

In this study, we are seeking to design and implement a family-based multimedia educational intervention using the telephone-tracking method (tele-nursing) to improve the quality of life and self-efficacy in patients with myocardial infarction.

## Research hypotheses

Considering the main study purpose, some hypotheses will be considered according to the opinions provided by a panel of experts in cardiovascular diseases, health education, and nursing. Some hypotheses will be:

After the intervention, quality of life is better in the intervention group compared with the control group.

After the intervention, the general self-efficacy is higher in the intervention group than the control group.

## Phase I: designing the multimedia and tele-nursing software curriculum

At this phase, the intervention will be designed and the target audience will be identified based on the review of literature, similar studies, and information collected from questionnaires. After the panel of experts’ confirmation, the educational content prepared by researchers will be formulated based on three main themes and presented to the audience. The educational program consists of several sections.

**The first section** will include a training session with 10-member groups. The educational contents will be provided through multimedia training (audio, video, image, and animation) about patients’ lifestyle, nutrition, and care. This session will be conducted as a one-day workshop in 2–3 hours for patients and one of their active family members. The active family member is defined as the primary caregiver, who spends more time with the patient. The educational course will be conducted at a coordinated date and time in the ward where the patients are hospitalized.

**The second section** will be carried out using question/ answer format; at the end of the workshop, a session will be devoted to answer the participants’ questions about the educational content.

**In the third section**, the participants will be provided with educational files (in CD), so that they can review the covered contents at home.

**The fourth section** will be the telephone intervention. At the end of this session, the participants’ phone numbers will be collected and the researcher will contact them once a week for one month. Details of the educational intervention program are presented in ***Table 1***.

**Table 1:** Multimedia educational intervention using the telephone-tracking method (tele-nursing).


PROGRAM	INTERVENTION CONTENT	PLACE AND METHOD OF PRESENTATION	AUDIENCE

Multimedia educational program	Introduction to exercise for patients with heart diseases; the type and level of activities Introduction to medication program, drug effects, medications’ side-effects and dosage in heart patients Introduction to diet and foods’ type, amount, and frequency in heart patients	In the form of a one-day workshop during 2–3 hours Using audio, video, image, and animation In the ward where the patients were hospitalized	Patients and their active family members

Question and answer	Questions and answers about the workshop’s content	Question/answer and discussion at the end of the workshop	Patients and their active family members

Presenting the educational file	Educational file in the form of CD	Providing the participants with the educational CD at the end of workshop	Patients and their active family members

Telephone-tracking (tele-nursing)	Verbal encouragement of the health-care behaviors related to the educational contents provided at the workshop or in the training CD	1month (once a week) The researcher coordinated the contact time with the patients and their family members	Patients and their active family members


## Experts’ team

The content and visual validity of the Educational program will be measured, and the comments received from the panel of experts. The panel of experts will be included three professionals from the cardiovascular disease and two experts from the field of nursing and one expert from the health education.

## Phase II: Implementation of educational intervention

In this step, a randomized controlled clinical trial will design to assess the efficacy of prepared protocol. Participants randomly will allocate into two groups. In one experimental group we will implement our designed protocol, and control group will not receive any intervention, but they follow as same as experimental group.

Participants will be selected based on the inclusion criteria and randomly divided into the intervention and control groups. After completing the written informed consent forms, the individuals will complete the pre-test questionnaires and the intervention group will receive the researcher-made educational package. During this time, the control group will not receive any education, but only the routine nursing care. At the post-test stage, that is one month after the intervention, all participants will be asked to complete the questionnaires and the collected data will be analyzed.

## Study environment and population

The patients hospitalized in the critical care units (CCU) of the hospitals affiliated to Shahrekord University of Medical Sciences will be considered for the intervention.

## Study sample

The target participants will include all patients (and their active family members) admitted to CCUs of hospitals affiliated to Shahrekord University of Medical Sciences. The sample size was calculated as 60 (30 participants in each group) based on the previous studies and with respect to the type I error of 0.05 and the test power of 0.80.

## Sampling method

Random sampling’s method will apply to select the participants.

Participants will be divided into the intervention (receiving the training program) and control (not receiving the training program) groups according to the random number table.

## Inclusion criteria

- Definitive diagnosis of the myocardial infarction by a cardiovascular specialist for the first time;
- Admission to the CCU in hospitals affiliated to Shahrekord University of Medical Sciences;
- Age range of 45 to 88 years;
- Possibility of making telephone communication;
- Ability to speak Farsi without having difficulty in speaking, hearing, and seeing;
- Access and ability to use the computer in order to read the educational files at home;

## Exclusion criteria

- Refusal to continue participating in the study;
- Patient’s death during the research;
- Prolonged hospital stay due to the disease complications;

## Data collection method

### 1. Minnesota Quality of Life Questionnaire

This questionnaire was designed by Rector in 1984 to determine the impact of treatment on quality of life in patients with heart failure (30). This questionnaire is the most common tool used to assess the patients’ quality of life in research studies (31). The Minnesota Questionnaire assesses the patients’ perception from the heart failure effects on the physical, social, and psychological aspects of their lives. This scale contains 21 items dealing with the physical (N = 13), psychological (N = 4), and socio-economic (N = 4) aspects (32). Each question contains 6 criteria ranging from zero (the best situation) to five (the worst condition). The attainable scores can range from zero to 105; higher total scores indicate greater impact of the disease on the patients’ quality of life (i.e., lower quality of life). A change of 5 scores or more shows a significant improvement in severity of symptoms and quality of life. This questionnaire has had high reliability and validity compared to other similar questionnaires and achieved a Cronbach’s alpha of above 0.9 in all previous studies (33).

### 2. Sherer’s General Self-efficacy Questionnaire

It has 17 items on general self-efficacy and measures the individuals’ beliefs about their own ability to overcome different situations. The questionnaire is designed based on a five-point Likert scale (strongly disagree: 1 score, strongly agree: 5 scores). The maximum and minimum attainable scores in this questionnaire are 85 and 17, respectively. The validity and reliability of the questionnaire have been confirmed by Iranian and international studies. The reliability coefficient of this questionnaire was reported as 76% and 79% by Gutman’s split-half method and by Cronbach’s alph, respectively (34).

## Data analysis

We will apply X2 and independent T test to analysis our data by using SPSS version 21.

## Outcome measures

1. Quality of life includes physical, psychological, economic, and social dimensions, which will be measured by the Minnesota Quality of Life Questionnaire.
2. General self-efficacy, which includes the individual’s beliefs about their ability to overcome different situations, will be measured by the Sherer General Self-efficacy Questionnaire.

## Discussion

The present study provides useful data for designing a family-based multimedia educational intervention using the telephone-tracking method (tele-nursing) to improve the quality of life and self-efficacy in patients with myocardial infarction.

Asgari et al. examined the effect of family-based education on laboratory parameters of patients after myocardial infarction and showed that the family-based educational model improved the laboratory parameters of patients with myocardial infarction [2].

Vahedian et al. reported that implementing a family-based empowerment model was practically feasible for patients with myocardial infarction and improved their quality of life [13]. Behzad et al. showed that the telephone follow-up empowerment program was effective in promoting self-efficacy in self-care behaviors among elderly people with hypertension [4]. Diane Inman et al. also investigated the impact of tele-nursing on patients with prostate surgery. Their results showed that 47% of the intervention group members needed less care facilities than the controls [9].

In the present training package, the educational media will be incorporated to enhance education outcomes; so, it can be effective in supporting patients with cardiovascular diseases. It can also reduce their medical and treatment costs. We suppose this program has capacity to integrate into the professionally health care guidelines, so that it can help medical, nurses and health care providers pay attention to the important role of the heart patient health, especially during stay in CCU. The strategies of this program could be important and cost effective, and therefore we hope that the success of such a program is a step forward in improving cardiovascular patient’s health status. Furthermore, the strategy used in this protocol can be applied to provide care and implement tele-nursing in other chronic diseases.

## Abbreviations

MI: Myocardial Infarction

CCU: Critical Care Units
